# A Review of Tunnel Field-Effect Transistors: Materials, Structures, and Applications

**DOI:** 10.3390/mi16080881

**Published:** 2025-07-29

**Authors:** Shupeng Chen, Yourui An, Shulong Wang, Hongxia Liu

**Affiliations:** Key Laboratory of Wide Bandgap Semiconductor Materials, Faculty of Integrated Circuit, Ministry of Education, Xidian University, Xi’an 710071, China; 23111213718@stu.xidian.edu.cn (Y.A.); slwang@xidian.edu.cn (S.W.); hxliu@mail.xidian.edu.cn (H.L.)

**Keywords:** tunnel field-effect transistor (TFET), ultra-low-power electronics, subthreshold swing (*SS*), band-to-band tunneling (BTBT)

## Abstract

The development of an integrated circuit faces the challenge of the physical limit of Moore’s Law. One of the most important “Beyond Moore” challenges is the scaling down of Metal-Oxide-Semiconductor Field-Effect Transistors (MOSFETs) versus their increasing static power consumption. This is because, at room temperature, the thermal emission transportation mechanism will cause a physical limitation on subthreshold swing (*SS*), which is fundamentally limited to a minimum value of 60 mV/decade for MOSFETs, and accompanied by an increase in off-state leakage current with the process of scaling down. Moreover, the impacts of short-channel effects on device performance also become an increasingly severe problem with channel length scaling down. Due to the band-to-band tunneling mechanism, Tunnel Field-Effect Transistors (TFETs) can reach a far lower *SS* than MOSFETs. Recent research works indicated that TFETs are already becoming some of the promising candidates of conventional MOSFETs for ultra-low-power applications. This paper provides a review of some advances in materials and structures along the evolutionary process of TFETs. An in-depth discussion of both experimental works and simulation works is conducted. Furthermore, the performance of TFETs with different structures and materials is explored in detail as well, covering Si, Ge, III-V compounds and 2D materials, alongside different innovative device structures. Additionally, this work provides an outlook on the prospects of TFETs in future ultra-low-power electronics and biosensor applications.

## 1. Introduction

With the continuous advancement of CMOS integrated circuit (IC) technology, key process dimensions in semiconductor manufacturing process have been continuous scaled down with the Moore’s Law. However, traditional Metal-Oxide-Semiconductor Field-Effect Transistors (MOSFETs) face the 60 mV/decade (mV/dec) physical limitation of subthreshold swing (*SS*), which is governed by the thermal emission process and the Fermi–Dirac distribution of carriers. This constraint creates a significant trade-off between power consumption and performance, making it challenging to enhance performance without increasing power consumption. Furthermore, the scaling down of traditional MOSFETs causes the short-channel effects, resulting in degraded device performance [1]. The recent appearance of Tunnel Field-Effect Transistors (TFETs) shows a great potential to solve the 60 mV/dec limitation problem faced by CMOS devices. Unlike traditional MOSFETs, TFETs leverage a band-to-band tunneling carrier injection strategy as the primary transport mechanism, enabling carriers to traverse the channel barrier via quantum tunneling rather than the thermal emission process in MOSFETs. Consequently, TFETs bypass the Fermi–Dirac distribution limitation, delivering enhanced switching characteristics at reduced voltages and positioning them as promising candidates for ultra-low-power applications [2,3].

The concept of TFETs dates back to the 1970s, initially featuring a homojunction structure, where both the source and drain were processed by same semiconductor material [4]. The first tunnel field-effect transistor (TFET) was fabricated in 2004 by Joerg Appenzeller, featuring a carbon nanotube as the channel. It achieved a small *SS* of 63 mV/dec [5]. In 2007, Woo Young Choi et al. successfully fabricated a 70 nm n-channel silicon-based TFET, achieving a low *SS* drop to 52.8 mV/dec at room temperature; this result paved the way for the development of sub-60 mV/dec *SS* in silicon-based TFETs [6]. In 2008, Tejas Krishnamohan et al. proposed a double-gate TFET design, which enhanced electric field control and increased tunneling probability by incorporating a second gate, thereby improving device performance [7]. This structure demonstrated higher on-currents and reduced leakage currents, showing promise in initial experiments. In 2011, Wei Cao introduced the p-n-i-n structure with a pocket, further enhancing TFET performance through a simulation analysis method [8]. In 2012, a team from Seoul National University developed an L-shaped channel TFET, with an original intention to increase the tunneling area by extending the point tunneling mechanism to line tunneling mechanism [9]. In 2013, Munetaka Noguchi fabricated an InGaAs TFET with a switching ratio of 10^6^; this result greatly promotes the application of TFET as a logic switch [10]. In the same year, M. J. Kumar proposed and simulated a dopingless tunnel field-effect transistor (DL-TFET) [11] and B. Ghosh proposed and simulated a Junctionless Tunnel Field Effect Transistor (JL-TFET) [12], which simplified the fabrication process of TFET by avoiding the doping process of abrupt tunneling junction. In 2015, Deblina Sarkar fabricated a TFET by using a two-dimensional material MoS_2_ [13], paving the way for extensive exploration into 2D-material-based TFETs, which has sparked a research boom on 2D-material-based TFET structures. In 2017, Shupeng Chen et al. proposed a novel Symmetric U-shaped TFET with bidirectional current flow and proper performance [14], which can simplify the TFET digital design. In 2020, Seungho Kim et al. utilized bulk BP (black phosphorus) and monolayer BP to construct a homojunction 2D-TFET [15]. In 2021, Clarissa Convertino et al. fabricated an InGaAs/GaAsSb TFET by using nearly standard silicon processes, which provided a valuable reference for the fabrication process of TFETs [16]. In 2022, Shupeng Chen proposed a novel TFET (QB-TFET) based on quasi-broken gap energy band alignment [17], achieving an ultra-low *SS* and an exceptionally high *I_on_*/*I_off_* ratio.

In order to review the development history of TFETs more clearly, the remaining discussion of this review article is divided into three major parts. The first part mainly discusses the experimental research results of TFETs; this section shows the feasible ways to manufacture TFETs by using the current state-of-the-art technology and reveals the benchmark of the TFETs performance with experiment result. The second part focuses on simulation research results of TFETs; this section indicates the theoretical potential of TFET performance and shows the directions for TFET structure and material optimization in the future. The third part briefly introduces the potential applications and popular areas of TFETs in the future.

## 2. Experiment Research Works on TFETs

### 2.1. Si/Ge-Based TFETs

**Figure 1:**
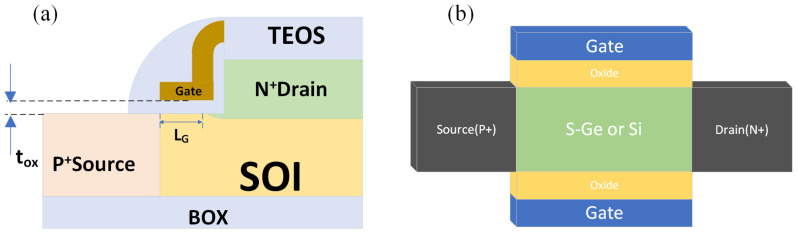
(**a**) Schematic structure of the silicon-based TFET. (**b**) Schematic of the double-gate TFET.

Silicon-based TFETs are among the earliest proposed and experimentally studied TFET devices. Woo Young Choi et al. developed a TFET using Silicon-On-Insulator (SOI) technology [6]. This work represents the first experimental observation of a silicon-based TFET achieving sub-60 mV/dec *SS*, with a minimum *SS* of 52.8 mV/dec at room temperature. The on-state and off-state currents were measured as 12.1 µA/µm and 5.4 nA/µm with *V_D_* = 1 V, respectively. Although the *SS* was lower than that of MOSFETs, the switching ratio (*I_on_*/*I_off_*) was inferior to that of MOSFETs. As illustrated in Figure 1a, the source and drain were located on the side and top of the “SOI channel”, with the gate positioned above the “SOI channel”, separated by an oxide layer. The study highlighted the influence of gate oxide thickness and SOI thickness on *SS* values, showing a reduction in *SS* with thinner oxide and SOI layers. Experimental data indicated that with a gate oxide thickness (*t_ox_*) of 2 nm and an SOI thickness (*t_SOI_*) of 70 nm, the *SS* remained below 60 mV/dec.

However, silicon faces some challenges as a TFET material due to its large and indirect bandgap. On the one hand, during band-to-band tunneling, electrons have to pass through a relatively thick triangular tunneling barrier. On the other hand, electrons must simultaneously abide by the conservation of energy and momentum. This will lead to a low tunneling rate and result in a low tunneling current. Additionally, traditional silicon-based TFET fabrication requires heavy doping, which is still difficult to achieve with the state-of-the-art processing techniques. Krishna Mohan et al. proposed a Si/Ge heterojunction structure combined with an innovative double-gate (DG) design, as illustrated in Figure 1b. Their experimental results demonstrated a DG, strained-Ge (s-Ge) TFET achieving a record on-state current (*I_on_*) of approximately 300 µA/µm and an *SS* of around 50 mV/dec [7].

**Figure 2:**
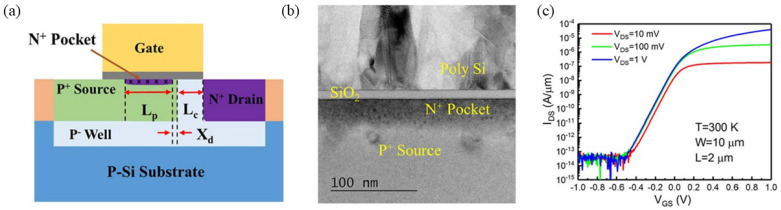
(**a**) Schematic structure of the Si line tunneling TFET. (**b**) The TEM image of the Si line tunneling TFET. (**c**) Transfer characteristic curves of the Si line tunneling TFET [18]. Reproduced with permission from ref. [18].

A novel silicon-based line tunneling TFET has recently emerged through the work of Weijun Cheng et al. [18]. This innovative structure, presented in Figure 2a,b, features an N⁺ pocket layer integrated between source and gate terminals to facilitate carrier tunneling along the entire interface. The enlarged tunneling cross-section substantially enhances tunneling current generation. The experimental results revealed a minimum subthreshold swing (*SS_min_*) of 69 mV/dec and an average subthreshold swing (*SS_avg_*) of 80 mV/dec at room temperature. Additionally, the device achieved a high *I_on_* of 40 µA/µm at *V_DS_* = 1 V while maintaining an off-state current (*I_off_*) below 1 pA/µm, as shown in Figure 2c.

### 2.2. Heterojunction TFETs Based on III-V Compounds

**Figure 3:**
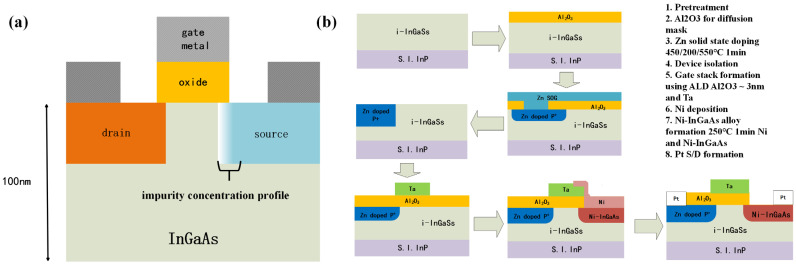
(**a**) Schematic structure of the In_0.53_Ga_0.47_As TFET. (**b**) Schematic fabrication process of the In_0.53_Ga_0.47_As TFET.

III-V compound materials, owing to their good electrical properties, have also been one of the major topics of discuss in the fabrication of TFETs. Compared to Si/Ge, the direct and small bandgap of some III-V compounds can give a lower tunneling barrier for band-to-band tunneling of carriers. Additionally, their lower effective masses for electrons and holes, combined with extremely high electron mobility and steep density of states (DOS), significantly enhance tunneling efficiency. From the fabrication perspective, III-V compounds can be readily integrated into silicon-based processes through heteroepitaxy, offering greater flexibility for designing high-performance and low-power integrated circuits. Munetaka Noguchi et al. fabricated a planar heterojunction TFET by doping the source region of 100 nm In_0.53_Ga_0.47_As/S.I.InP wafers with Zn [19]. Figure 3a illustrates the structure of the device, and Figure 3b describes the specific fabrication process of the device. The solid-phase diffusion of Zn formed a steep-profile and defect-free p^+^/n source junction due to Zn diffusion characteristics in InGaAs, significantly enhancing TFET performance. This device achieved a minimum *SS* of 64 mV/dec with *I_on_* of 10^−6^ A/μm, *I_off_* of 10^−12^ A/μm, and *I_on_*/*I_off_* of 10^6^.

Additionally, nanowire TFETs are a popular research field, especially in III-V compounds TFETs. Due to the high surface-to-volume ratio of their nanowire structures, the gate can comprehensively envelop the channel and provide a strong control. This “Gate-All-Around” structure effectively suppresses short-channel effects and minimizes leakage currents, thereby enhancing device performance such as *I_on_*/*I_off_* [20]. Moreover, nanowire TFETs are well suited for compact nanoscale fabrication processes and exhibit strong compatibility with existing technologies [21]. This makes them particularly advantageous for achieving high-density integrated circuits applications. Anil W. Dey et al. developed a nanowire heterojunction TFET, which exhibited relatively higher *I_on_* [22]. This device utilized a GaSb/InAs (Sb) heterojunction with a type II broken band alignment and was fabricated through metal–organic vapor phase epitaxy. As shown in 

**Figure 4:**
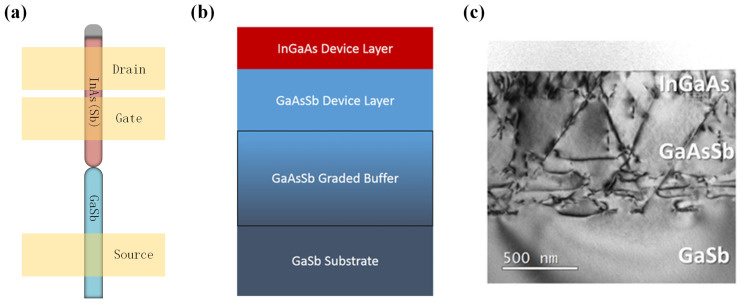
(**a**) Schematic of the GaSb/InAs(Sb) heterojunction TFET. (**b**) Schematic of the InGaAs/GaAsSb NW-TFET. (**c**) TEM images of the InGaAs/GaAsSb tunnel junctions [25]. (**b**,**c**) are reproduced with permission from ref. [25].

Figure 4a, the structure of the InAsSb/GaSb NW-TFET is presented. However, studies have revealed that a significant number of dislocations are generated at the heterojunction interface between InAs and GaSb, which adversely affects carrier tunneling [23,24].

**Figure 5:**
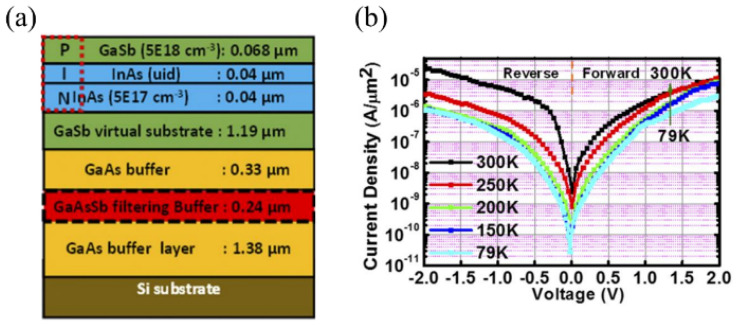
(**a**) Structure of the InAs/GaSb NW-TFET. (**b**) J-V characteristics of the InAs/GaSb NW-TFET [35]. Reproduced with permission from ref. [35].

To address the issue of dislocations at the heterojunction interface, Ryan M. et al. fabricated an In_0.8_Ga_0.2_As/GaAs_0.3_Sb_0.7_ NW-TFET using metal-organic chemical vapor deposition (MOCVD) [25]. In Figure 4b, the growth structure of the tunnel diode is illustrated. The fabrication process involved first growing a GaAsSb buffer layer on a GaSb substrate, followed by the growth of an InGaAs/GaAsSb functional layer, effectively reducing dislocations caused by lattice mismatches. However, according to TEM images, as shown in Figure 4c, a significant number of threading and misfit dislocations were observed at the InAs/GaSb and InGaAs/GaAsSb interfaces despite the relatively small lattice mismatch. This phenomenon may be attributed to the MOCVD growth process, as the use of gaseous reactants in MOCVD can introduce impurities. Furthermore, during material growth, MOCVD may result in Ga carryover effects due to methyl exchange reactions between trimethylindium (TMIn) and trimethylgallium (TMGa), potentially causing Ga atoms to migrate to the upper layers and leading to dislocation formation [23,26,27,28,29,30,31,32] and elemental exchange [33,34]. Such issues can have a significant negative impact on achieving high-quality tunnel junction interfaces. In 2018, Jheng-Sin Liu et al. used solid-source molecular beam epitaxy (MBE) to achieve heterogeneous integration of an InAs/GaSb NW-TFET using a 200 nm strained GaAs_1−y_Sb_y_ dislocation-filter buffer layer [35]. Figure 5a shows the growth structure, while Figure 5b presents the *J-V* characteristics of the fabricated tunnel junction. At room temperature, the tunnel junction exhibited a tunneling current of 5 × 10^−5^ A/μm^2^ at 2 V reverse bias under room temperature.

Beyond InGaAs/GaSb, the InGaAs/InAs heterojunction has also been explored for TFET design. Xin Zhao et al. developed a nanowire TFET featuring a heterojunction between p⁺-InGaAs and intrinsic InGaAs, with a 2 nm i-InAs/8 nm i-In_0.7_Ga_0.3_As “notch” layer inserted to reduce the tunneling barrier and enhance drive current [36]. Process optimizations included improved ALD chamber conditioning to ensure uniform Al_2_O_3_ deposition, followed by five digital etch cycles post-RIE to refine nanowire diameter to 20 nm, improving surface quality. Rapid thermal annealing was also employed to passivate interface traps, thereby suppressing trap-assisted tunneling (TAT) effects. The device demonstrated a temperature-insensitive *SS*, achieving 53 mV/dec at *V_DS_* = 0.3 V at room temperature and maintaining 37 mV/dec at *V_DS_* = 0.05 V down to 77 K.

**Figure 6:**
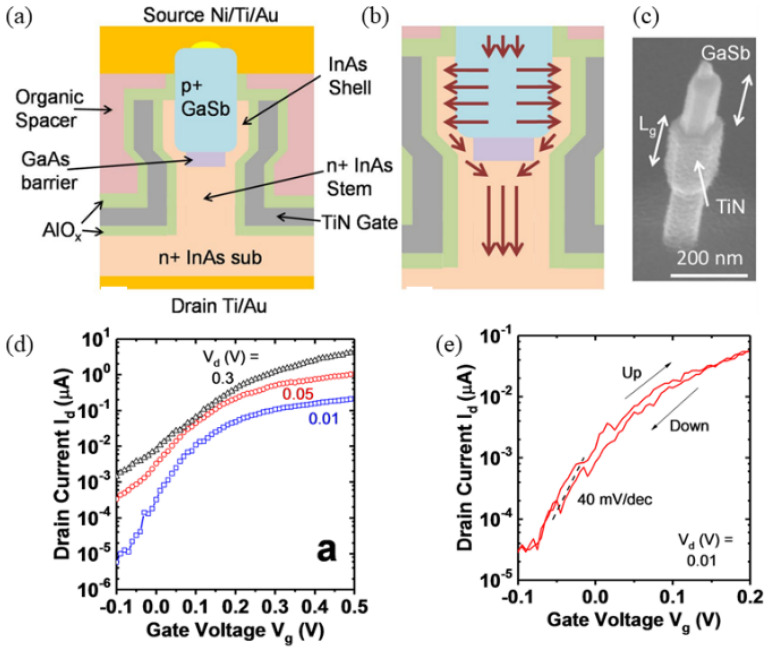
(**a**) Structural depiction of the VGAA C-S TFET. (**b**) Enhanced magnification highlighting electron trajectories within the device. (**c**) Scanning electron micrograph (SEM) of the nanowire (NW) segment positioned above the gate electrode following selective digital removal of the InAs shell layer. (**d**,**e**) *I_D_–V_G_* characteristics [37]. Reproduced with permission from ref. [37].

The adoption of a Gate-All-Around (GAA) structure can enhance the performance of Nanowire TFETs by further improving the surface-to-volume ratio. In 2019, T. Vasen et al. fabricated Vertical Gate-All-Around Nanowire GaSb-InAs Core-Shell n-Type Tunnel FETs (VGAA C-S TFET) [37]. The structure and corresponding SEM images are shown in Figure 6a–c. The testing results, illustrated in Figure 6d,e, demonstrate an *SS_min_* of 40 mV/dec at *V_D_* = 10mV. The device achieves a current drive of 39.4 μA/μm, normalized to a GaSb core diameter of 35 nm, at *V_D_* = 0.3 V and *V_G_* = 0.5 V. Recent research on nanowire TFETs continues to attract significant interest [38,39]. For instance, the newly proposed ferroelectric tunnel field-effect transistor utilizes a nanowire structure [40], which enables frequency doubling without generating additional harmonics, making it highly suitable for signal transmission applications.

**Figure 7:**
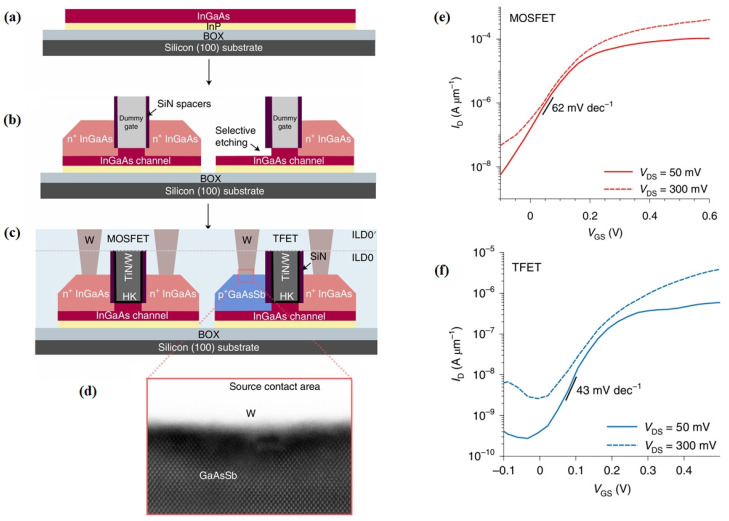
(**a**–**c**) Schematics for the fabrication process steps of the InGaAs MOSFET and the InGaAs/GaAsSb TFET. (**d**) High-resolution TEM image assessing the crystalline quality in the GaAsSb exposed region. (**e**) Transfer characteristic of the InGaAs MOSFET. (**f**) Subthreshold characteristic for a TFET with L_G_ = 25 nm and W_FIN_ = 25 nm [16]. Reproduced with permission from ref. [16].

In 2021, Clarissa Convertino et al. fabricated an InGaAs MOSFET and an InGaAs/GaAsSb TFET using near-standard silicon manufacture processes [16]. The structure and main fabrication process of the device are shown in Figure 7a–d. Unlike pure MOSFET processes, the epitaxial regrowth of electrode contact zones was conducted in two separate steps, forming n-type and p-type regions independently. MOSFET electrodes and TFET drain terminal comprised epitaxially grown n^+^ Sn-doped In_53_Ga_47_As (1 × 10^19^ cm^−3^), while the TFET source was formed from p^+^ Zn-doped Ga_0.5_As_0.5_Sb (4 × 10^19^ cm^−3^), lattice-matched to InGaAs. This design enabled clear differentiation between TFETs and MOSFETs at the device level, facilitating flexible hybrid logic block design. Measurements showed that at room temperature, with *V_DS_* = 50 mV, the minimum *SS* for the GaAsSb TFET at *L_G_* = 25 nm and *W_FIN_* = 25nm was 43 mV/dec. The MOSFET counterpart measured a minimum *SS* of 62 mV/dec under similar conditions, with electrical stability demonstrated across varying temperatures (Figure 7e,f). This study confirmed the feasibility of manufacturing TFETs using standard silicon-CMOS-compatible processes, paving the way for designing ultra-low-power hybrid circuits.

### 2.3. TFETs Based on 2D Materials

In recent years, many TFETs have been fabricated using two-dimensional (2D) materials including Molybdenum disulfide (MoS_2_), black phosphorus (BP), tungsten diselenide (WSe_2_), etc. [41,42,43,44]. Two-dimensional semiconductors exhibit excellent gate controllability due to their ultra-thin layered structure. However, their bandgap can be influenced by the quantum confinement effect depending on the number of layers. The fabrication of 2D TFETs must therefore consider both material selection and the number of layers. TFETs based on 2D materials are categorized as homojunction or heterojunction TFETs, with heterojunctions further divided into 2D/2D and 2D/3D types.

**Figure 8:**
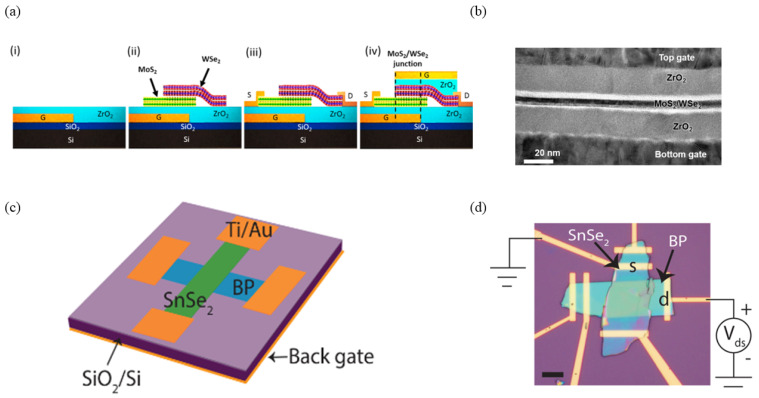
(**a**) Schematics for the fabrication process steps of the MoS_2_/WSe_2_ TFET. (**b**) Cross-sectional TEM image of the MoS_2_/WSe_2_ TFET [47]. (**c**,**d**) Schematic illustration and optical image of the BP/SnSe_2_ heterojunction TFET [48]. (**a**,**b**) are reproduced with permission from ref. [47]. (**c**,**d**) are reproduced with permission from ref. [48].

**Figure 9:**
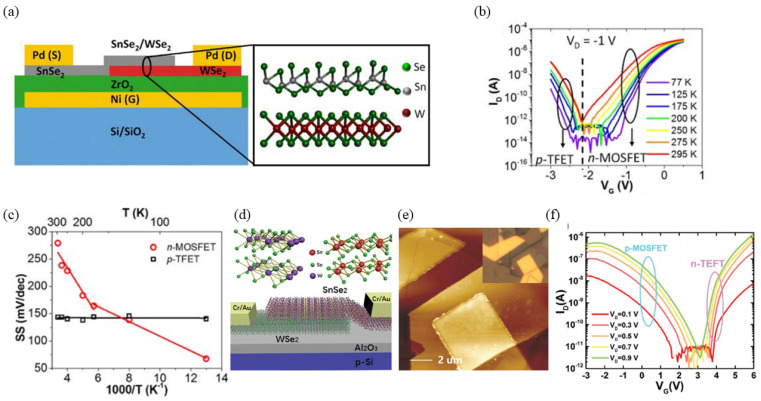
(**a**) Device structure of the WSe_2_/SnSe_2_ TFET. (**b**) Transfer characteristic of the WSe_2_/SnSe_2_ TFET. (**c**) The subthreshold swing (*SS*) of both devices varies with temperature [49]. (**d**) Schematic of the WSe_2_/SnSe_2_ TFET. (**e**) Atomic force microscopy (AFM) topography of the fabricated device. (**f**) Transfer characteristic of WSe_2_/SnSe_2_ heterostructure [50]. (**a**–**c**) are reproduced with permission from ref. [49]. (**d**–**f**) are reproduced with permission from ref. [50].

Heterojunction TFETs leverage staggered or broken band alignments, generating sharper band profiles than those achievable through doping modulation alone. Therefore, they enhance significant enhancement in both on-current and subthreshold steepness. For example, vertical heterojunction TFETs using materials such as WSe_2_ can exhibit band offset transitions from a staggered gap (type-II) to a broken gap (type-III) under high positive back-gate voltages, inducing tunneling at channel biases [45,46]. In 2015, Tania Roy et al. constructed a vertical 2D/2D heterojunction TFET using MoS_2_ and WSe_2_, mechanically transferred onto a Si/SiO_2_ substrate, with top-gate stacking to enable dual-gate control (Figure 8a,b) [47]. In the same year, Rusen Yan et al. developed a BP (black phosphorus)/SnSe_2_ heterojunction TFET, further validating tunneling mechanisms (Figure 8c,d) [48]. In 2016, Tania Roy incorporated ZrO_2_ as a gate dielectric to enhance gate control and reduce *SS* in WSe_2_/SnSe_2_ vertical TFETs, achieving a minimum *SS* of 100 mV/dec, though still above 60 mV/dec (Figure 9a–c) [49]. In 2017, Xiao Yan fabricated a WSe_2_/SnSe_2_ vertical TFET with an ultra-thin Al_2_O_3_ gate dielectric, achieving a minimum *SS* of 37 mV/dec and an average *SS* of approximately 80 mV/dec, with a maximum on-current of ~1.5 μA and an *I_on_*/*I_off_* exceeding 10^6^ (Figure 9d–f) [50].

**Figure 10:**
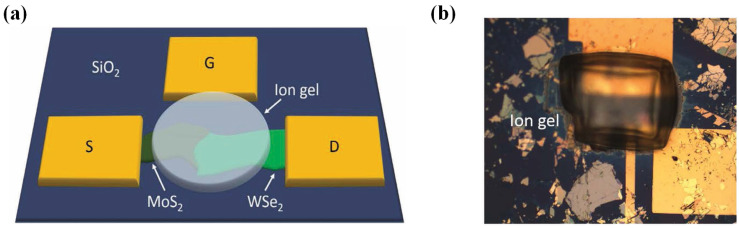
(**a**) WSe_2_/MoS_2_ van der Waals heterostructure TFET schematic. (**b**) Optical micrograph post ion-gel top-gate deposition. [51]. Reproduced with permission from ref. [51].

To further enhance the performance of 2D/2D heterojunction TFETs, Hyun Bae Jeon et al. introduced the use of an ion gel as a top-gate dielectric in MoS_2_/WSe_2_ heterojunction TFETs [51]. Figure 10a shows the structure of the device, and Figure 10b presents the microscope image of the device. The ion gel dielectric exhibited high capacitance, creating strong electric fields in small-channel regions, enhancing gate control and reducing *SS*. This approach also enabled modulation from type-II to type-III (broken gap) band alignment, facilitating band-to-band tunneling (BTBT) with steep *SS* and high *I_on_*/*I_off_*. This device achieved a minimum *SS* of 36 mV/dec, with an *I_on_*/*I_off_* of 10^6^.

**Figure 11:**
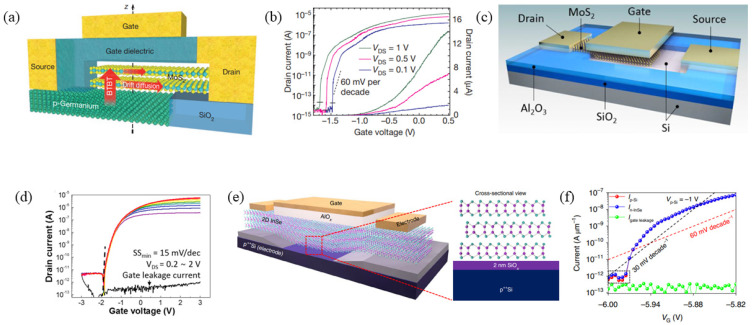
(**a**) Cross-sectional schematic of MoS_2_/Ge heterojunction TFET. (**b**) Corresponding transfer characteristics (*I_D_*-*V_G_*) [13]. (**c**) Device structure of the MoS_2_/Si TFET. (**d**) *I_DS_*-*V_GS_* transfer characteristics of the MoS_2_/Si TFET [52]. (**e**) Schematic of an n-InSe/p^++^Si 2D/3D HJ-TT. (**f**) Transfer characteristic of 2D/3D HJ-TT [53]. (**a**,**b**) are reproduced with permission from ref. [13]. (**c**,**d**) are reproduced with permission from ref. [52]. (**e**,**f**) are reproduced with permission from ref. [53].

In addition to using 2D/2D heterojunctions, vertical tunneling field-effect transistors can also incorporate 2D/3D heterojunctions. Deblina Sarkar et al. transferred MoS_2_ onto p-Ge to form a TFET structure, as illustrated in Figure 11a. This device utilized double-layer MoS_2_ synthesized through chemical vapor deposition (CVD), chosen for its high electron affinity compared to other 2D materials. Meanwhile, Ge, as a 3D material, exhibited lower electron affinity than other Group III-V compounds and IV elements. The experimental results (Figure 11b) revealed an average *SS* of 46.4 mV/dec at room temperature. However, further studies indicated that the formation of a Ge oxide layer during the process limited carrier tunneling probability [13]. In 2018, Gwang Hyuk Shin et al. fabricated a vertical MoS_2_/Si TFET using a trilayer MoS_2_ and heavily doped Si (10^19^ cm^−3^), with Al_2_O_3_ serving as the gate dielectric, as depicted in Figure 11c. Figure 11d displays the transfer characteristic curves of the device, and the device demonstrated a minimum *SS* of 23 mV/dec and an *I_on_*/*I_off_* exceeding 10^7^ [52]. In 2021, Jinshui Miao and collaborators constructed a 2D/3D TFET using n-type 2D InSe and heavily p-doped 3D silicon. Their process involved etching square windows on the SiO_2_/p^++^Si substrate to expose the underlying p^++^Si layer, followed by transferring mechanically exfoliated 2D InSe flakes onto the exposed surface. The device structure is shown in Figure 11e. Testing at room temperature with *V_p-Si_* = −1 V yielded a minimum *SS* of 6.4 mV/dec and an average *SS* of 30 mV/dec, with a maximum on-current density of *I_p-Si_* = 0.3 µA /µm. A comparison with InSe MOSFETs revealed a minimum *SS* of ~100 mV/dec (Figure 11f) [53].

**Figure 12:**
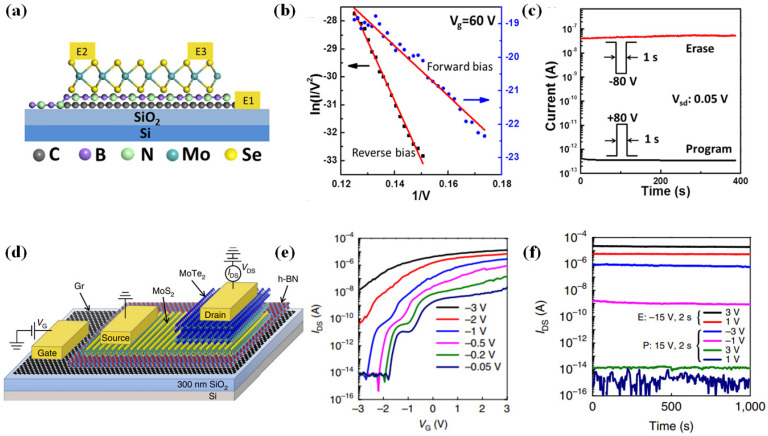
(**a**) Device structure of the Cr/hBN/MoS_2_ TFET. (**b**) ln(*I/V^2^*) − *1/V* curves. (**c**) Retention characteristic of the memory [54]. (**d**) Structure schematic of the Cr/hBN/MoS_2_/MoTe_2_ TFET. (**e**) *I_DS_*-*V_G_* transfer curves. (**f**) Retention characteristics of programmed and erased states under varying read bias conditions [55]. (**a**–**c**) are reproduced with permission from ref. [54]. (**d**–**f**) are reproduced with permission from ref. [55].

Hexagonal boron nitride (h-BN), a wide-bandgap two-dimensional (2D) material, is frequently employed as a dielectric or tunneling barrier in 2D material-based memory devices. Ruiqing Cheng et al. proposed a novel tunneling field-effect transistor (TFET) structure by inserting a thin layer of h-BN between two tunnel-capable 2D materials, resulting in a Cr/hBN/MoS_2_ TFET (Figure 12a) [54]. When the thickness of the h-BN layer is relatively small (~3.2 nm), the device exhibits direct tunneling characteristics. However, for thicker h-BN layers (~9.6 nm), the device demonstrates clear Fowler–Nordheim tunneling (FNT) behavior. A linear relationship in the ln(*I/V^2^*) − *1/V* plot is observed above 5.8 V (Figure 12b), confirming the triangular barrier-induced FNT mechanism, with an extracted barrier height of h-BN of approximately 3.43 eV. Under FNT conditions, the tunneling current can be effectively modulated via gate control of the Fermi level in the graphene electrode. The device achieves an on/off current ratio of 5 × 10^3^. Furthermore, it demonstrates a high rectification ratio of 7 × 10^5^ and excellent non-volatile memory performance, with a program/erase (P/E) ratio exceeding 10^5^ and retention time over 400 s (Figure 12c). This work is a significant advancement, offering new insights into the development of 2D-TFETs and opening avenues toward high-performance 2D rectifiers and memory devices.

Building upon this design, the team further improved the device by replacing the MoS_2_ channel with a MoS_2_/MoTe_2_ heterostructure, forming an asymmetric Cr/hBN/MoS_2_/MoTe_2_ TFET (Figure 12d) [55]. Under positive drain bias (*V_DS_* > 0), the device behaves like a conventional p–n junction dominated by thermionic emission, whereas under negative bias, it transitions into a TFET regime. In this mode, the gate voltage (*V_G_*) effectively modulates the tunneling barrier width, enabling efficient control over the tunneling process. The experimental results reveal an impressive on/off current ratio up to 6 × 10^8^ (Figure 12e), a five-order-of-magnitude improvement over the previous structure. Additionally, the rectification ratio is enhanced to 10^8^, and the memory performance is significantly improved with a P/E ratio > 10^9^ VD and retention time exceeding 1000 s (Figure 12f). The device also exhibits excellent photoresponse, suggesting strong potential for 2D material-based optoelectronic and memory-integrated systems.

**Figure 13:**
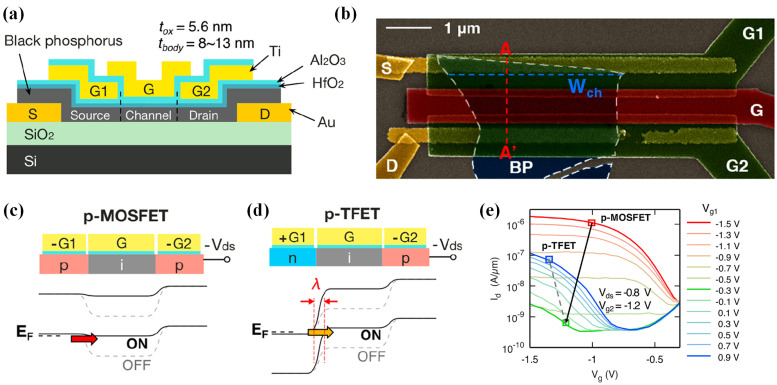
(**a**) BP RED-TFET architecture. (**b**) False-colored SEM image of the BP RED-TFET. (**c**) p-MOSFET schematic and band diagram. (**d**) p-TFET operational schematic and band diagram. (**e**) ransfer characteristics under p-configuration [56]. Reproduced with permission from ref. [56].

Besides heterostructures, 2D materials can be employed to fabricate homojunction TFETs, avoiding trap-related issues arising from contact between different materials and thus enhancing device reliability. Unlike heterostructures that rely on van der Waals (vdWs) linkages and multiple stacking layers, homojunctions are less challenging for modern manufacturing processes. In 2019, Peng Wu et al. developed a homojunction TFET using black phosphorus (BP), exploiting electrostatic doping through different gates. The device structure is depicted in Figure 13a,b. Applying opposing bias polarities to gates (G1, G2) and the middle top gate (G) created tunnel p-i-n or n-i-p junctions; for instance, a positive *V_G1_* doped the BP base n-type, while a negative *V_G1_* induced p-type doping (Figure 13c,d). This approach enabled the creation of p-type or n-type 2D-MOSFETs and TFETs. Tests revealed a minimum *SS* of 170 mV/dec and an on-current (*I_on_*) of 0.6 μA/μm (Figure 13e) [56].

**Figure 14:**
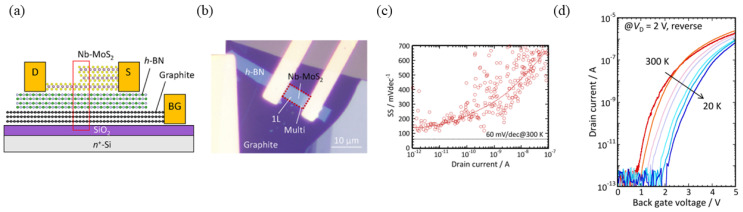
(**a**) Schematic the TM-homojunction Nb-doped MoS_2_ TFET. (**b**) Optical micrograph of the TM-homojunction Nb-doped MoS_2_ TFET. (**c**) *SS* of the Nb-doped MoS_2_ TFET as a function of temperature (**d**) Temperature dependence of transfer characteristics of Nb-doped MoS_2_ TFET [57]. Reproduced with permission from ref. [57].

In addition to utilizing gate voltage for electrical doping, the unique thickness-dependent electrical properties of 2D materials can also be exploited to fabricate TFETs. In 2023, Tomohiro Fukui et al. utilized homojunctions formed by MoS_2_ layers of varying thickness to create TFETs. Figure 14a shows the structure of the device, and Figure 14b presents the microscope image of the device. Due to changes in bandgap (*E_G_*) with layer thickness, they grew Nb-doped p^+^ MoS_2_ with a hole concentration of 2 × 10^19^ cm^−3^ via chemical vapor transport (CVT). Mechanical exfoliation, however, caused S vacancies on the MoS_2_ surface, leading to an n-type transition due to higher electron concentrations from vacancies than Nb-induced hole concentrations. Consequently, a homojunction was formed between MoS_2_ layers of different thicknesses. Measurements showed a minimum *SS* of 140 mV/dec at room temperature (Figure 14c,d) [57].

**Figure 15:**
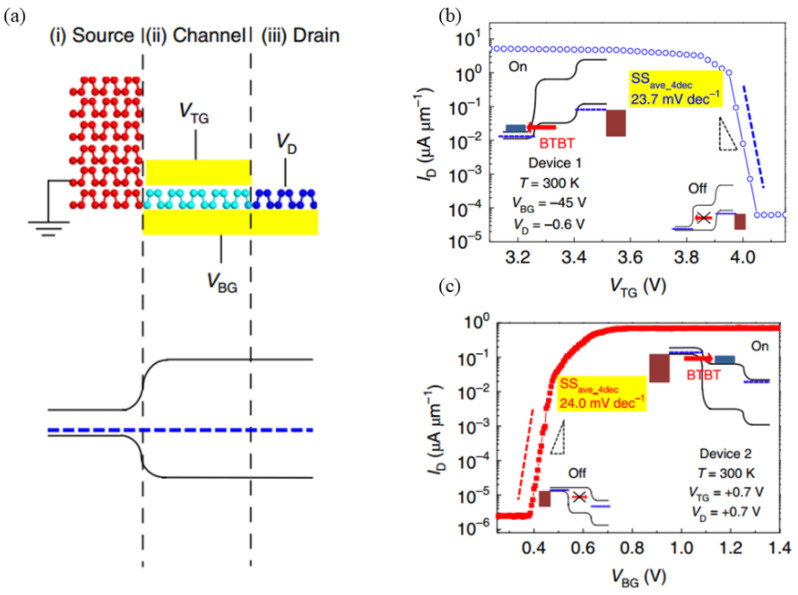
(**a**) Schematic structure and energy band diagrams of BP NHJ-TFET at source, channel, and drain regions. (**b**) p-type transfer characteristics for Device 1 with indicated subthreshold swing (*SS*). (**c**) n-type transfer characteristics for Device 2 with corresponding *SS* [15]. Reproduced with permission from ref. [15].

In 2020, Seungho Kim et al. utilized bulk BP and monolayer BP to construct a homojunction TFET. The source region consisted of bulk BP, the channel region was formed by a monolayer of BP, and the drain was capped with ultra-thin hBN and graphene (Figure 15a). Representative transfer characteristics of two BP NHJ TFET devices are presented in Figure 15b,c. Device 1 incorporated a 285 nm SiO_2_ bottom-gate dielectric and 10 nm hBN top-gate dielectric, with channel dimensions *L* ≈ 0.7 μm and *W* ≈ 1 μm. This device exhibited p-type TFET operation, achieving *SS_ave,4dec_* < 60 mV/dec. At *V_D_* = −0.6 V, Device 1 surpassed all previously reported TFETs (including n-type variants) in both *SS_ave,4dec_* and *I_60_* metrics, with *I_60_* ≈ 65 μA/μm, aligning with the preferred operational range (1–10 μA/μm). The *I_D_*-*V_TG_* transfer curve (10 nm hBN gate dielectric) yielded *SS_ave,4dec_* ≈ 23.7 mV/dec and *I_60_* ≈ 65 μA/μm. Device 2 employed a 3 nm hBN bottom-gate dielectric and 5 nm hBN top-gate dielectric, with *L* ≈ 0.5 μm and *W* ≈ 1 μm. Under n-type operation at *V_D_* = +0.7 V, this configuration achieved record-low *SS_ave,4dec_* along with the highest *I_60_* reported for sub-thermionic TFETs. The measurement results (3 nm hBN dielectric) indicated *SS_ave,4dec_* ≈ 24.0 mV/dec and *I_60_* ≈ 0.054 μA/μm. Notably, both devices required substantially lower switching voltages (Device 1: Δ*V_TG_* = 0.15 V; Device 2: Δ*V_BG_* = 0.2 V) compared to state-of-the-art MOSFETs (Δ*V_G_* ≥ 0.7 V), signifying significantly reduced power consumption in BP NHJ-TFETs [15]. However, BP’s poor stability in air remains a challenge for practical applications [58].

### 2.4. Carbon Nanotubes TFET

Carbon nanotubes (CNTs), with their unique cylindrical geometry, have also emerged as a material of choice for TFET fabrication [59,60]. Their natural cylindrical shape allows for partial or full gate wrapping, enhancing gate control [60]. The CNT-TFET was first proposed by J. Appenzeller et al., utilizing band-to-band tunneling induced by applying a negative back-gate voltage and a positive top-gate voltage, resulting in band bending and an *SS* around 40 mV/dec [5]. When a negative voltage is applied to the top gate, the device behaves like a traditional MOSFET with thermionic emission dominating and the *SS* exceeding 60 mV/dec.

**Figure 16:**
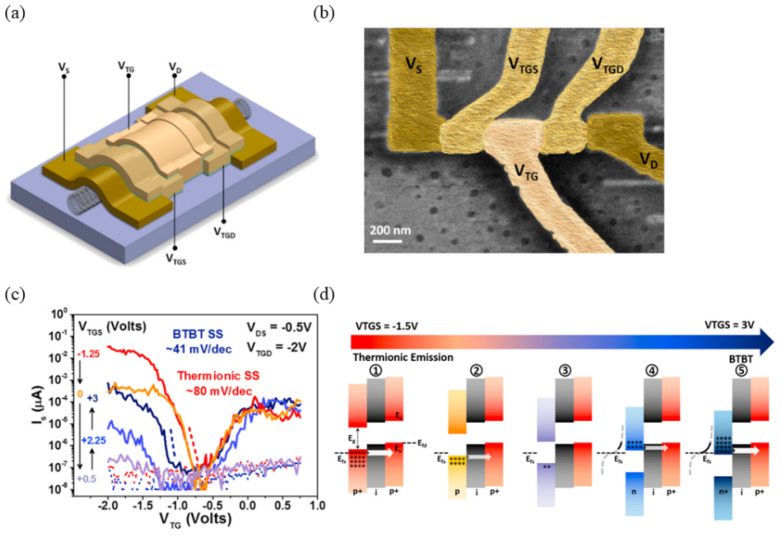
(**a**) Electrode configuration with voltage bias scheme. (**b**) Scanning electron micrograph of fabricated CNT triple-gate device (scale bar: 200 nm). (**c**) Transfer characteristics under varying top-gate voltage conditions. (**d**) Energy band diagrams at different gate biases [61]. Reproduced with permission from ref. [61].

In 2021, Chin-Sheng Pang and colleagues developed a CNT TFET using a multi-gate structure, as shown in Figure 16a. And Figure 16b shows an SEM image of the device. Similar to the aforementioned BP-based TFET [57], the device utilized *V_TGS_* control to adjust transistor states (Figure 16d). As *V_TGS_* increased, the material beneath gradually transitioned from p-type to n-type, forming an n-i-p tunnel junction. Tests showed a minimum *SS* of 41 mV/dec at room temperature, with a maximum tunneling current of 100 nA (Figure 16c) [61]. While CNT TFETs hold promise, further improvements are necessary to fully realize their potential.

**Figure 17:**
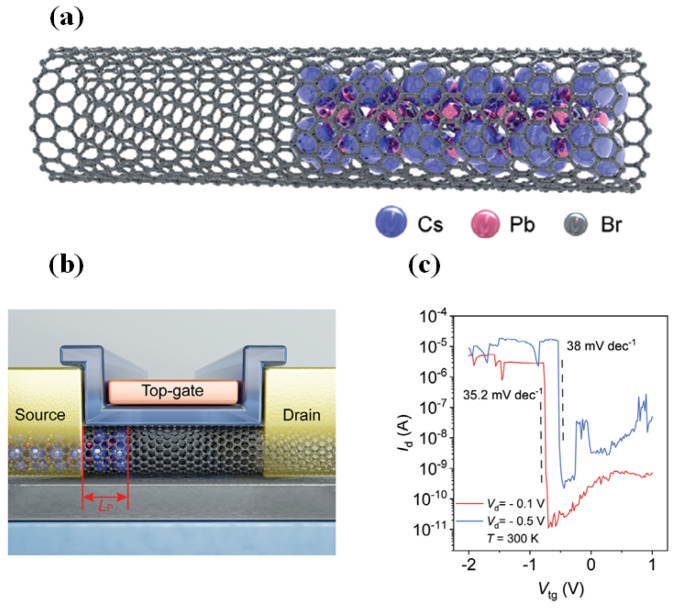
(**a**) Schematic of the coaxial CsPbBr_3_/CNT. (**b**) Schematic of the CsPbBr_3_/CNT heterojunction FET. (**c**) Transfer characteristics of the CsPbBr_3_/CNT heterojunction FET [63]. Reproduced with permission from ref. [63].

Enhancing tunneling efficiency with suppressed leakage necessitates band-alignment engineering via controlled doping in CNTs. Chongwu Zhou et al. explored surface doping by adsorbing potassium atoms via chemical vapor deposition. However, this approach suffered from poor long-term stability [62]. To address this, Maguang Zhu et al. proposed an internal cavity doping method using encapsulated perovskites instead of surface modifiers (Figure 17a,b) [63]. This strategy greatly improved device reliability, with HAADF-STEM confirming no degradation of the perovskite crystal structure even after 30 days. Electrical characteristics remained stable, achieving *SS* = 35.2 mV/dec, an *I_on_* of 4.94 μA per tube, and an *I_on_/I_off_* ratio exceeding 10^5^ at room temperature and *V_D_* = −0.1 V (Figure 17c).

Table 1 presents an experimental comparison of key electrical parameters for the aforementioned TFET devices. Overall, 2D materials exhibit a promising trend of surpassing 3D materials in the field of TFETs. It is worth noting that 2D materials are still in the early stages of development. Therefore, further simulations and experimental research are necessary to comprehensively explore and establish the design-level correlations between 2D material systems, device design strategies, and synthesis/manufacturing technologies. This approach will ultimately enable CMOS-level performance at the nanoscale.

**Table 1:** Experimental comparison of electrical parameters for fabricated TFETs.

Device Name	*SS* (mV/dec)	*I_on_* (μA/μm)	*I_on_/I_off_*	*V_d_* (V)	Ref.
SOI Si-TFET	52.8	12.1	∼2.2 × 10^3^	1.0	[6]
Si/Ge DG TFET	~50	~300	-	-	[7]
Si Line Tunneling TFET	69	40	>10^7^	1.0	[18]
InGaAs TFET	64	1.0	10^6^	-	[19]
InGaAs/GaAsSb TFET	43	-	-	0.05	[16]
InAs/GaSb NW-TFET	-	5 × 10^−5^ (A/μm^2^)	-	2.0	[35]
WSe_2_/MoS_2_ TFET	36	100	10^6^	−1.0	[51]
Cr/hBN/MoS_2_ TFET	-	-	5 × 10^3^	-	[54]
Cr/hBN/MoS_2_/MoTe_2_ TFET	-	-	6 × 10^8^	−1.0	[55]
WSe_2_/SnSe_2_ TFET	37	1.5	>10^6^	-	[50]
MoS_2_/Ge TFET	46.4	-	-	-	[13]
MoS_2_/Si TFET	23	-	>10^7^	-	[52]
InSe/Si TFET	6.4	0.3	-	−1.0	[53]
BP RED-TFET	170	0.6	-	0.8	[56]
Nb-doped MoS_2_ TFET	140	-	-	2.0	[57]
BP Homojunction TFET	23.7–24.0	-	-	0.6/0.7	[15]
CNT Triple Gate TFET	41	0.1	-	-	[61]
CsPbBr_3_ CNT TFET	35.2	4.94	>10^5^	−0.1	[63]

## 3. Simulation Research Works on TFETs

Given that TFET has become one of the international hot research topics in the post-Moore era, performance optimization of TFET in terms of structures, materials, working mechanisms, etc., needs to be maximized excavation and research. This results in a large number of researchers using simulation methods to analyze and study the performance of TFETs and has produced a large number of research results till now. This section will focus on the simulation research results on TFETs; the research findings in this part indicate the theoretical potential of TFET performance and show the directions for TFETs structure and material optimization in the future. Table 2 summarizes the key performance parameters of the TFETs to be discussed below.

**Table 2:** Simulated performance metrics of advanced TFET architectures.

Device Name	*SS* (mV/dec)	*I_on_* (μA/μm)	*I_on_/I_off_*	*V_d_* (V)	Ref.
NC-LTFET	18.3	0.24	-	-	[64]
S-TFET	45	-	10^5^	0.5	[65]
SUTFET	15.2	13.5	4.4 × 10^6^	0.5	[14]
HTG-TFET	36.59	7.02	-	0.2	[66]
OGDL-TFET	20.6	75.5	9 × 10^13^	0.5	[67]
DF-TFET	18.2	58.8	-	-	[68]
SNPJL-TFET	20	54.3	1.45 × 10^14^	0.5	[69]
QB-TFET	4.9	921	-	0.5	[17]

### 3.1. p-n-i-n TFET

**Figure 18:**
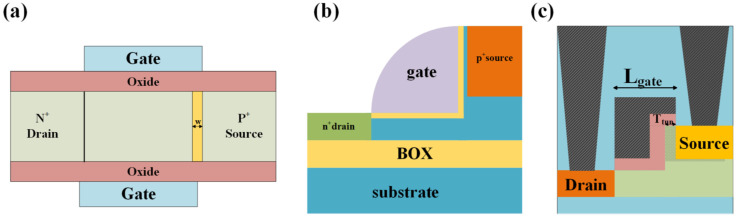
(**a**) p-n-i-n TFET schematic showing layer configuration and contacts. (**b**) Schematic of L-shaped TFET configuration. (**c**) Fabrication process flow.

To improve the tunneling probability of the traditional p-i-n structure, Wei Cao et al. proposed the p-n-i-n configuration [8]. This modified architecture, shown in Figure 18a, inserts a thin n-doped interlayer at the p-i tunneling junction to enhance the tunneling electric field (*E_y_*). A progressive increase in the doping density in the interlayer was experimentally demonstrated to enhance overall device conductance. Simulations quantifying gate oxide influence indicated significantly suppressed threshold voltage fluctuations induced by oxide variations, thereby establishing a foundational framework for further TFET optimization.

### 3.2. L-Shaped TFET

The L-shaped TFET was pioneered by Sang Wan Kim et al. [9], with its structural configuration depicted in Figure 18b. To enhance band-to-band tunneling (BTBT) efficiency, minimizing the tunneling barrier width (*W_t_*) between *E_V_* and *E_C_* is essential, given the exponential relationship between tunneling probability and *W_t_* [70]. Conventional TFETs exhibit *W_t_* variation modulated by gate voltage (*V_G_*), as it is governed by junction depletion dynamics. At low *V_G_* values, the significant *W_t_* dimension results in diminished tunneling probability, yielding gradual switching transitions and elevated subthreshold swing (*SS*) values. Channel length reduction further degrades performance due to increased off-state current (*I_off_*) from thermal emission, manifesting punch-through currents analogous to MOSFET behavior [71]. In contrast, L-shaped TFETs maintain a fixed *W_t_* determined by the *L_i_* dimension. This configuration achieves substantially steeper *SS* values compared to conventional designs. Crucially, whereas conventional TFETs restrict effective tunneling to nanometer-scale regions beneath the gate electrode, thereby limiting on-state current (*I_on_*) [72], L-shaped TFETs establish tunneling orthogonally to the channel direction (Figure 18b). This geometric advantage significantly expands the effective tunneling cross-section, enabling simultaneous improvement in both *SS* steepness and *I_on_* magnitude.

Sang Wan Kim et al. continued to explore the fabrication process of an L-shaped TFET and successfully produced devices with improved performance [73], as shown in Figure 18c. Experimental characterization reveals that the L-shaped TFETs have a less ideal *I_off_* compared to simulation results. But even though this is a less-ideal result, an *I_on_/I_off_* ratio of 5 orders of magnitude is achieved by the experimental results at *V_d_* = 0.05V. Thus, the L-shaped TFET still has great potential in applications of ultra-low-power circuits [74].

**Figure 19:**
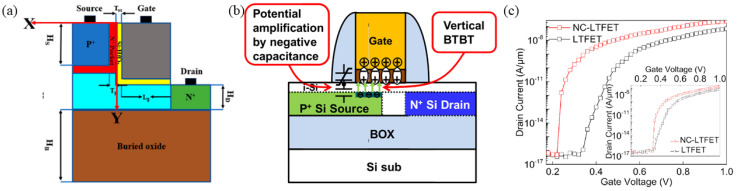
(**a**) The cross-section view of NC-LTFET. [64] (**b**) Schematic of NCTFET [75]. (**c**) The transfer characteristic curve of NC-LTFET and LTFET [64]. (**a**,**c**) are reproduced with permission from ref. [64]. (**b**) is reproduced with permission from ref. [75].

In 2022, Zhang, H et al. optimized the original L-shaped TFET design by proposing the Negative Capacitance LTFET (NC-LTFET) (Figure 19a) [64]. Inspired by the p-n-i-n structure [8], an n^+^ pocket layer was introduced at the p^+^ source. Additionally, drawing on Masaharu Kobayashi et al.’s research on NC-TFETs [75] (Figure 19b), a ferroelectric gate oxide layer was incorporated between the gate and the channel. According to Kobayashi’s findings, using ferroelectric materials in this configuration creates negative capacitance, generating an electric field that facilitates carrier tunneling. Si (SiO_2_-doped hafnium oxide) was employed as the gate oxide, with the manufacturing process for the ferroelectric materials (Si) detailed in reference [76]. The simulations yielded the transfer characteristic curves of the NC-LTFET (Figure 19c). The NC-LTFET demonstrated an *SS* of 18.3 mV/dec over a drain current range from 4 × 10^−17^ to 1 × 10^−9^ A/µm. Additionally, its large *I_on_* of 2.4 × 10^−7^ A/µm at *V_GS_* = 1 V suggests strong driving capability in digital circuits. Its small *I_off_* of 4 × 10^−17^ A/µm at *V_GS_* = 0 V also indicates minimal static power consumption in the off state. Consequently, the NC-LTFET is a highly promising candidate for low-power applications and merits further investigation.

### 3.3. U-Shaped TFET

**Figure 20:**
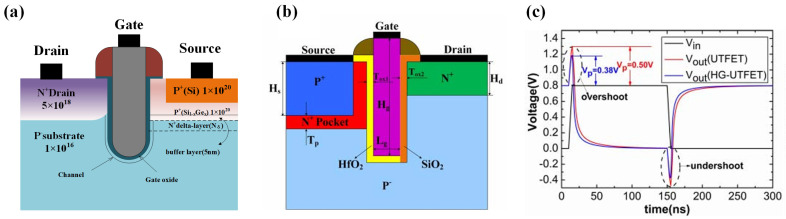
(**a**) Comparative SiGe-UTFET configurations with/without n⁺-delta layer. (**b**) Schematic of the HG -UTFET. (**c**) Transient response of TFET-based inverters demonstrating signal overshoot/undershoot [79]. (**b**,**c**) are reproduced with permission from ref. [79].

In 2014, Wei Wang et al. proposed the U-shaped TFET (UTFET), with its structure illustrated in Figure 20a [77]. Unlike planar TFETs, the UTFET channel is recessed into the substrate, transforming the lateral p-i-n configuration into a vertical direction, significantly enhancing tunneling efficiency and improving device performance [78]. Further optimizations were made, such as adding an additional heavily doped n-type auxiliary tunneling barrier layer at the source tunneling junction to increase tunneling efficiency [8]. The use of a heavily doped p-type germanium source and a silicon/germanium heterojunction as the tunneling junction further increased the tunneling efficiency compared to LTFETs [9]. This work compared five TFET configurations—Ge-UTFET, SiGe-UTFET, Si-UTFET, planar Si-TFET, and planar SiGe-TFET—by evaluating their transfer characteristics. The Ge-UTFET demonstrated significantly superior current drive capability relative to both Si-UTFET and planar Si-TFET under *V_G_* = 0.7V bias. Substituting the Ge source with Si_0.6_Ge_0.4_ substantially reduced *I*_on_ and *I*_off_—an effect principally attributed to SiGe’s wider bandgap (0.84 eV vs. Ge’s 0.67 eV). Crucially, all devices achieved optimized switching at *V_D_* = 0.7V, exhibiting an average subthreshold swing of 60 mV/dec with drain current modulation spanning over six orders of magnitude.

In 2017, Wei Li et al. introduced an improvement to the U-shaped TFET by proposing the heterogeneous gate UTFET (HG-UTFET) [79], with its structure shown in Figure 20b. This design aimed to address the issue of large Miller capacitance in TFETs. The Miller capacitance significantly impacts the frequency response of analog circuits and the delay characteristics of digital circuits [80,81,82]. When present, the Miller capacitance amplifies the effective capacitance transferred from the input to the output by a factor of (1 + *A_v_*), where *A_v_* is the voltage gain. In digital circuits, delay is proportional to capacitance, while in analog circuits, frequency is inversely proportional to capacitance. Thus, the presence of Miller capacitance negatively affects both digital and analog circuit performance. The large Miller capacitance in TFETs arises from their band-to-band tunneling conduction mechanism [83,84,85]. During gate voltage transitions from off- to on-state, the inversion layer of the TFET expands from the channel surface toward the source region, eventually covering the entire channel surface. Elevated gate–drain capacitance (*C_gd_*) results in the Miller capacitance being dominated by the gate–drain capacitance, with gate–source capacitance (*C_gs_*) contributing minimally. This behavior differs from MOSFETs, where the gate–source and gate–drain capacitances are similar before the formation of the inversion layer [86]. Once the inversion layer forms, the gate capacitance is mainly determined by the *C_gs_*.

To address the issue of large *C_gd_* in TFETs, researchers have proposed several solutions. Primarily, these include the use of heterogeneous gate dielectrics and gate–drain exposure methods [87,88,89,90]. The heterogeneous gate dielectric method involves dividing the TFET gate oxide layer into two sections: a high-k material is used near the source region to maintain strong gate control, while a low-k material is applied near the drain region to reduce gate-drain overlap capacitance [91]. Consequently, the left side of the HG-UTFET employs a high-k dielectric (HfO_2_), while the right side uses low-k SiO_2_. To demonstrate that the HG-UTFET offers a smaller Miller capacitance (*CM*) compared to the UTFET, Figure 20c shows that both inverters experience output signal overshoot/undershoot. HG-UTFET—demonstrates a ∼45.6% reduction in Miller capacitance compared to conventional UTFET. Furthermore, *CM* significantly affects TFET inverter delay characteristics, with HG-UTFET inverters exhibiting 30.7% shorter falling delay than UTFET implementations.

### 3.4. Symmetric Tunnel Field-Effect Transistor

**Figure 21:**
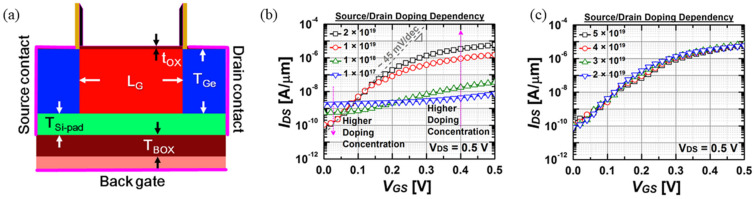
(**a**) Cross-sectional schematic of symmetric tunnel FET (S-TFET) highlighting structural layers. (**b**,**c**) *I_DS_-V_GS_* characteristics at *V_DS_ =* 0.5 V, showing source/drain doping effects: Source/drain doping concentration (**b**) lower than 2 × 10^19^ cm^3^ and (**c**) higher than 2 × 10^19^ cm^3^ [65]. Reproduced with permission from ref. [65].

Hyohyun Nam et al. pioneered the Symmetric TFET (S-TFET) featuring a unique p-i-p architecture [65]. As depicted in Figure 21a, this design employs n-type-doped silicon channels (*N*_Si_ = 10^16^ cm^−3^) with 5 nm thick lightly p-doped silicon pads (*N*_Si-pad_ = 10^15^ cm^−3^). Critical innovation lies in the 15 nm thick germanium source/drain regions (*N*_Ge_ = 2 × 10^19^ cm^−3^), where narrow-bandgap materials enhance *I_on_* performance. Contrasting conventional p-i-n configurations, the p-i-p topology facilitates bidirectional current flow while achieving sub-60 mV/decade subthreshold swing. The electrical characterization presented in Figure 21b,c establishes that under a bias voltage of *V_DD_* = 0.5 V, S-TFET devices with germanium source/drain doping concentrations exceeding 10^19^ cm^−3^ exhibit significantly enhanced electrical properties. The drive current consistently surpasses 5 μA/μm, while the *I*_on_/*I*_off_ ratios exceed 10^5^, accompanied by minimal subthreshold swing values approximating 45 mV/decade. Performance optimization reaches a plateau at the critical doping concentration *N*_Ge,S/D_ = 2 × 10^19^ cm^−3^, beyond which further increases in doping density yield no substantial improvement in either current magnitude or switching characteristics.

**Figure 22:**
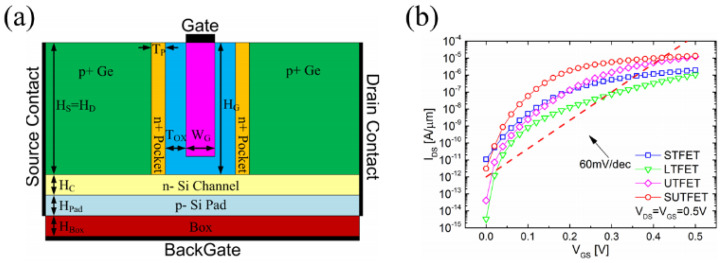
(**a**) Schematic of the SUTFET. (**b**) Transfer characteristic curves of S-TFET, L-TFET, U-TFET, and SUTFET [14]. Reproduced with permission from ref. [14].

In 2017, Shupeng Chen et al. proposed a novel Symmetric U-shaped Gate TFET(SUTFET) [14]. The structure, as shown in Figure 22a, features a gate resembling the shape of the letter “U” with p^+^ Ge source/drain regions flanking both gate sides. Two n^+^ pockets adjoining the gate enhance tunneling efficiency. The n-Si channel and p-Si pad are situated beneath the gate to create an *I_on_* channel for the device. Unlike conventional TFETs, SUTFET enables planar MOSFET-like bidirectional current flow, rendering it suitable for ultra-low-power ICs. Figure 22b presents simulation results comparing the transfer characteristic curves of the symmetric TFET (S-TFET) [65], L-TFET [73], U-TFET [77], and SUTFET [14], all derived using identical key device parameters, including the same n^+^ Si pocket height and width. The simulation results indicate that at room temperature with *V_DD_* = 0.5V, the min *SS* can reach 15.2 mV/dec, while the *I_on_* is 13.5 μA/μm, and the *I_on_*/*I_off_* is approximately 4.4 × 10^6^.

### 3.5. HTG-TFET

**Figure 23:**
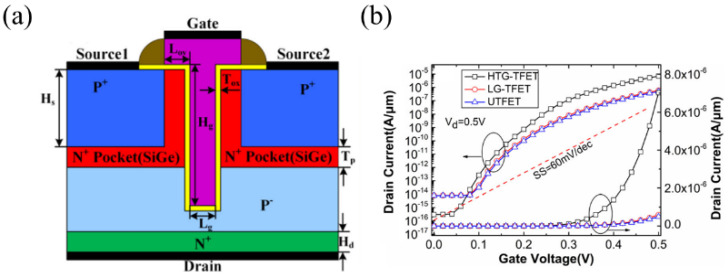
(**a**) Schematic structures of HTG-TFET. (**b**) Transfer characteristics of different devices [66]. Reproduced with permission from ref. [66].

Wei Li et al. innovatively integrated the L-shaped TFET [9] and SUTFET [14] architectures, proposing a T-gate heterojunction TFET (HTG-TFET), illustrated in Figure 23a [66]. This configuration expands the effective tunneling cross-section, significantly enhancing drive current. Under optimal simulation parameters, the HTG-TFET demonstrated superior performance, achieving an *I_on_* of 7.02 μA/μm and a minimum *SS* of 36.59 mV/dec at *V_G_* = 0.2 V (Figure 23b).

However, in practical TFET fabrication, heavy doping of the source and drain regions can lead to diffusion into the channel region, thereby affecting the steepness of the PN junction and altering the tunneling barrier width at the junction. Additionally, achieving uniform heavy doping is challenging due to the random fluctuations introduced by the ion implantation process. Researchers have studied the impact of doping variability on TFET performance, and generally, doping fluctuations can cause variations in the *I_on_* by up to two orders of magnitude [92,93,94], significantly affecting TFET performance. High-temperature annealing, often required during fabrication, further adds to the process cost.

### 3.6. DL-TFET and JL-TFET

**Figure 24:**
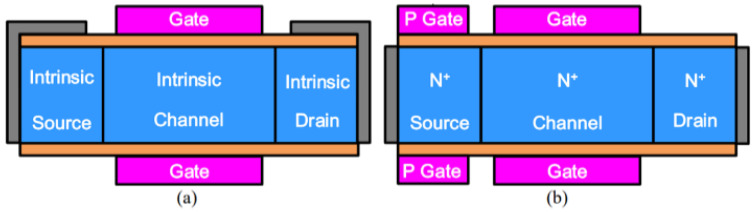
Schematic structures of (**a**) DL-TFET and (**b**) JL-TFET.

To address these issues, researchers proposed a dopingless TFET (DL-TFET) [11,95,96,97] and a junctionless TFET (JL-TFET) [12,98,99,100]. In dopingless TFETs, the source, channel, and drain regions are intrinsic materials, with the tunneling PN junction modulated by the work functions of the gate, source, and drain. Unlike conventional devices, the source and drain regions of dopingless TFETs form Schottky contacts instead of Ohmic contacts. By appropriately adjusting the work functions of the source and drain regions, holes accumulate in the source region, while electrons accumulate in the channel, forming a “pseudo-PN junction” through charge plasma effects. In contrast, all regions of the junctionless TFET are uniformly doped. The source region achieves inversion, and by tuning the metal work function of the source, holes accumulate to form a p-type region. Figure 24 illustrates the structural schematics of both dopingless and junctionless TFET devices. Existing studies have shown that these devices exhibit ultra-low *SS* and minimal *I_off_*. However, their primary limitation is the inability to achieve line tunneling, resulting in lower *I_on_* compared to conventional TFETs. Therefore, enabling line tunneling in DL-TFETs and JL-TFETs through effective strategies not only addresses fabrication challenges but also enhances the overall performance of TFETs.

**Figure 25:**
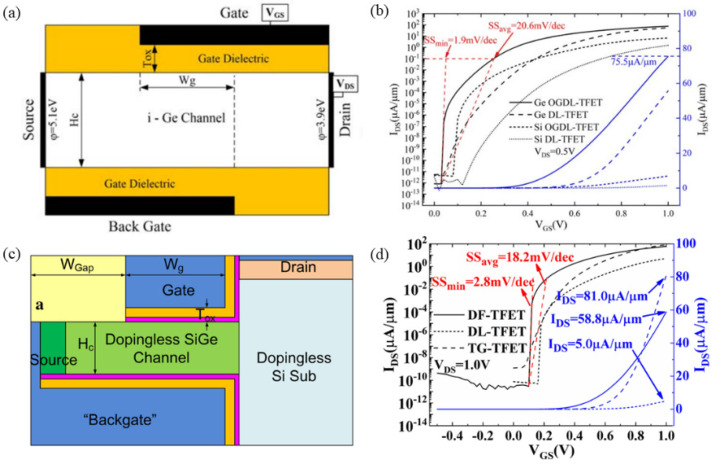
(**a**) Schematic of the OGDL-TFET. (**b**) Transfer characteristic curves of OGDL-TFET [67]. (**c**) Schematic of the DF-TFET. (**d**) Transfer characteristic curves of DF-TFET [68]. (**a**,**b**) are reproduced with permission from ref. [67]. (**c**,**d**) are reproduced with permission from ref. [68].

In 2019, Shupeng Chen improved the DL-TFET by proposing a germanium-based overlapping gate dopingless tunnel FET (OGDL-TFET) [67]. As illustrated in Figure 25a, this architecture positions gate and backgate electrodes on opposing channel surfaces. Differential work-function engineering induces energy band bending within their overlap region, establishing a line-tunneling junction via charge plasma principles. This line tunneling junction enables significantly increased *I_on_*/*I_off_* switching ratios through simultaneous *I_on_* amplification and *I_off_* suppression. The simulation results (Figure 25b) demonstrate that, at *V_DS_* = 0.5 V, the OGDL-TFET delivers *I_on_* = 75.5 μA/μm, *I_on_*/*I_off_* = 9 × 10^13^, *SS_min_* = 1.9 mV/dec, and *SS_avg_* = 20.6 mV/dec. Subsequently, in 2020, Shupeng Chen proposed a dopingless fin-shaped SiGe channel TFET (DF-TFET) [68]. This device also enables line tunneling for carriers, with its structure depicted in Figure 25c. The use of a fin structure in the DF-TFET significantly reduces the footprint compared to planar line tunneling TFETs. Consequently, the DF-TFET achieves an *I_on_* of 58.8 μA/μm, an *SS_min_* of 2.8 mV/dec, and an *SS_avg_* of 18.2 mV/dec (Figure 25d).

**Figure 26:**
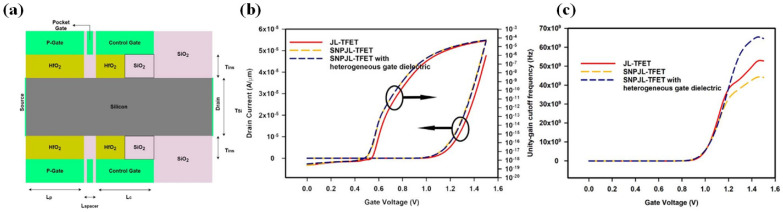
(**a**) Cross-section view of SNPJL-TFET with heterogeneous gate dielectric. (**b**) Transfer curve. (**c**) Unity-gain cutoff frequency (*F_T_*) of JL-TFET, SNPJL-TFET and SNPJL-TFET with heterogeneous gate dielectric [69]. Reproduced with permission from ref. [69].

In 2021, Sazzad Hussain et al. introduced a modified JL-TFET design termed SNPJL-TFET, which incorporated a pocket gate into the original structure, forming a p–n–i–n configuration to enhance tunneling probability (Figure 26a) [69]. Additionally, a heterogeneous gate dielectric stack was used, balancing gate control and parasitic capacitance. Compared to all-SiO_2_ or all-HfO_2_ gate stacks, the hybrid stack enhanced *C_g_* control while minimizing *C_gd_*, making the device better suited for high-frequency applications. Under *V_D_* = 0.5 V, the device achieved *I_on_* = 5.43 × 10^−5^ A, *I_on_/I_off_* = 1.45 × 10^14^, and *SS* = 20 mV/dec. It also exhibited a peak transconductance of 210 μS/μm, a cut-off frequency (*F_T_*) of 65.4 GHz, and a gain-bandwidth product (GBP) of 14.9 GHz (Figure 26b,c).

### 3.7. QB-TFET

**Figure 27:**
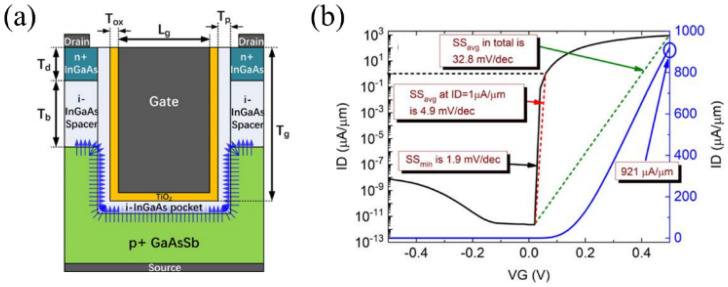
(**a**) Schematic of the QB-TFET. (**b**) Transfer characteristic curves of QB-TFET [17]. Reproduced with permission from ref. [17].

In 2022, Shupeng Chen et al. developed a quasi-broken gap aligned tunneling FET (QB-TFET) to optimize subthreshold swing and switching efficiency [17]. As illustrated in Figure 27a, to achieve high *I_on_*, the QB-TFET employs an InGaAs/GaAsSb heterojunction with quasi-broken gap energy band alignment, which improves the band-to-band tunneling rate. An intrinsic InGaAs spacer is incorporated between the n⁺ InGaAs drain and p⁺ GaAsSb source to minimize off-state leakage resulting from source-drain tunneling pathways. Additionally, TiO_2_ is used as the gate dielectric to improve the gate voltage control over the channel. Consequently, under a supply voltage of 0.5 V, the QB-TFET achieves a high *I_on_* of 921 μA/μm and an *SS_avg_* as low as 4.9 mV/dec (Figure 27b).

## 4. Applications of TFETs

**Figure 28:**
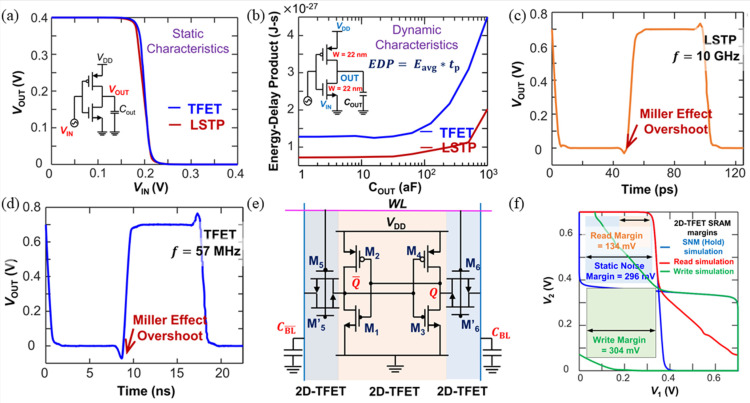
(**a**) Static characteristics of minimum-size 2D-TFET/LSTP inverters (*V_DD_* = 0.4 V). (**b**) Energy-delay product (EDP) comparison across capacitive loads. (**c**) LSTP and (**d**) 2D-TFET oscillatory waveforms (10 GHz vs. 57 MHz), noting Miller overshoot in (**d**). (**e**) Unidirectional current-addressed all-2D-TFET SRAM circuit. (**f**) Static noise margin analysis for hold/read/write operations (*V_DD_* = 0.7 V) [101]. Reproduced with permission from ref. [101].

TFETs, especially 2D-TFETs, offer significant advantages in circuit design due to their low power consumption. Arnab Pal et al. used Hspice simulations to analyze the performance, robustness, and power consumption of circuits like inverters, ring oscillators, and SRAMs designed with WTe_2_/MoS_2_-HJ-TFETs, comparing them to those based on 7 nm FinFET technology, as shown in Figure 28 [101]. Among these, the 2D-TFET inverter outperformed its 7 nm FinFET-based counterpart. Although both transistors demonstrate comparable transfer curves (Figure 28a), the 2D-TFET inverter achieves 48% higher voltage gain owing to enhanced drain current saturation (Figure 28b). For TFET-based ring oscillators, the oscillation frequencies reached 10 GHz and 57 MHz, with single-stage delays of 10 ps and 1.6 ns, respectively, indicating weaker performance compared to FinFET-based oscillators (Figure 28c,d). The designed 2D-TFET SRAM (Figure 28e) demonstrated relatively ideal performance, as shown in Figure 28f. The all-2D-TFET SRAM cell exhibits read, write, and hold margins of 133 mV, 304 mV, and 296 mV, respectively, at *V_DD_* = 0.7 V. Overall, these circuit modules performed well and can be readily integrated into more complex circuits.

**Figure 29:**
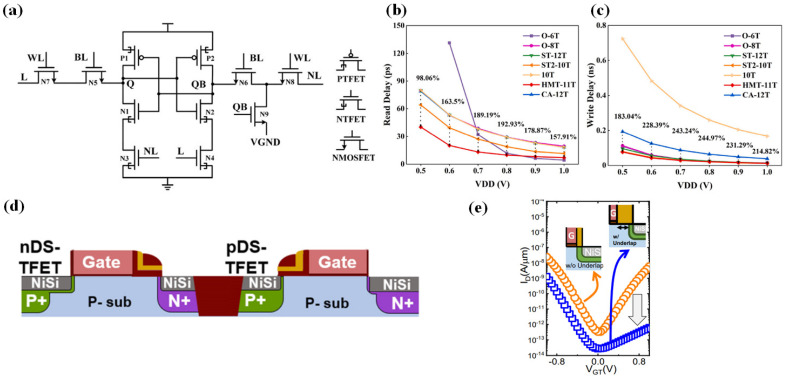
(**a**) Schematic of the HMT-11T SRAM cell. (**b**,**c**) Comparative read and write access latencies across SRAM cell architectures [102]. (**d**) Schematic structures of C-DS-TFETs. (**e**) Transfer curves of pDS-TFETs [103]. (**a**–**c**) are reproduced with permission from ref. [102]. (**d**,**e**) are reproduced with permission from ref. [103].

Recent progress has also been made in hybrid TFET-MOSFET circuit designs. Wenjuan Lu et al. proposed an 11-transistor (11T) SRAM architecture that integrates TFETs and MOSFETs (Figure 29a) [102]. In this design, the read path is implemented using MOSFETs, and an additional write-assist transistor is included, both contributing to significantly reduced read and write delays compared to traditional SRAM architectures (Figure 29b,c). This translates to much lower dynamic power consumption. The use of TFETs in the storage nodes lowers the minimum operating voltage, which, in turn, enhances read/write speed advantages over MOSFET-based SRAMs at reduced voltages. While all-TFET SRAMs suffer from increased static power due to forward p-i-n leakage at higher *V_DD_*, the 11T design mitigates this by using MOSFETs for write-access transistors, eliminating the forward p-i-n current and thereby lowering static power. This approach presents a viable route to low-power SRAM design.

In another breakthrough, Kaifeng Wang et al. achieved monolithic integration of a dopant-segregated TFET (DS-TFET) (Figure 29d) into a 300 mm CMOS baseline process [103]. The core innovation lies in leveraging silicide-induced dopant segregation effects. And the standard NiSi process forms a sharp doping gradient at the Si interface (SIMS reveals a gradient > 5 × 10^20^ cm^−3^/nm), improving junction abruptness by three orders of magnitude over conventional ion-implanted junctions. This effect, combined with a self-aligned gate architecture, enables high electric field tunneling at the gate edge, resulting in a 1000× increase in p-type TFET drive current. To suppress ambipolar leakage, a thick CMOS spacer was used as a hard mask to define a non-overlapped drain region, reducing the off-current to ~10^−13^ A (Figure 29e). This technology offers a practical path toward mass production of TFET-CMOS hybrid low-power circuits.

**Figure 30:**
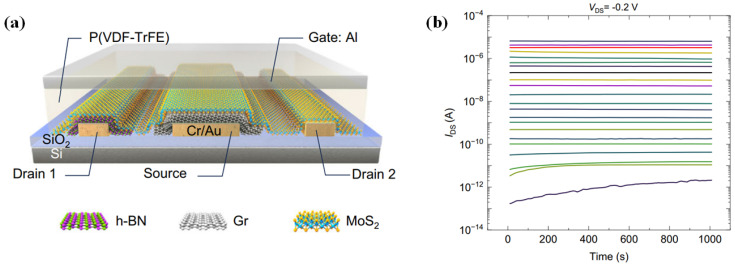
(**a**) Schematic of the MoS_2_-FeFET. (**b**) Retention characteristic of the memory [104]. Reproduced with permission from ref. [104].

As previously discussed, 2D-TFETs exhibit considerable promise for non-volatile memory (NVM) applications. In 2024, Guangdi Feng et al. introduced a vertical 2D-TFET utilizing a ferroelectric layer, with a MoS_2_/hBN/metal tunneling junction (Figure 30a) [104]. The ferroelectric polarization effectively modulates the Fermi level of MoS_2_, thereby tuning the tunneling current across the MoS_2_/metal interface. Inserting the hBN barrier not only suppresses the off-state current but also enhances the on-state current. This device achieved an exceptional on/off current ratio of 10^9^, with an on-current exceeding 20 µA and an off-current as low as 10−^14^ A. Moreover, the write energy was reduced to 0.16 fJ, demonstrating outstanding non-volatile memory characteristics and ultra-high multi-level storage capacity with 22 distinct stable states (Figure 30b). This work overcomes conventional contact injection limitations and provides a novel design paradigm for low-power, memory-in-computer systems.

**Figure 31:**
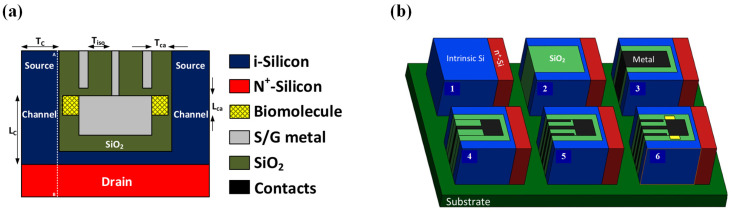
(**a**) A schematic cross-sectional view of the proposed DMDS-TFET biosensor structure. (**b**) Fabrication process steps (1–6) for realizing DMDS-TFET structure [105]. Reproduced with permission from ref. [105].

TFETs have extensive applications in sensors [105,106,107,108,109,110,111,112,113,114,115], with biosensors being a prominent example [105,106,107,108,109]. Compared to MOSFETs, TFETs offer better suitability for biosensing due to their shorter response time and lower leakage. In 2023, Iman Chahardah Cherik and Saeed Mohammadi designed a novel TFET-based biosensor with dual dopingless tunneling junctions (DMDS-TFET) [105]. The device functions similarly to DL-TFETs [11], as illustrated in Figure 31a. The sensor detects biomolecules by measuring changes in the dielectric constant within a cavity when biomolecules enter, thereby affecting device performance. Higher dielectric constants for biomolecules increase the probability of carrier tunneling and raise the *I_on_*. They also analyzed the device’s fabrication process (Figure 31b), which aligns with traditional CMOS technologies. Beyond biosensors, TFETs also find great potential in temperature sensors [112], pH sensors [113] and gas sensors [114,115] applications.

## 5. Conclusions

Experimental investigations have demonstrated that Si and Ge possess relatively wide bandgaps and high carrier effective masses, which limit the probability of band-to-band tunneling and consequently result in a low *I_on_*. This makes it challenging to achieve high-performance switching at low supply voltages [116]. Moreover, the fabrication of TFETs based on these materials typically requires heavy doping concentrations, leading to increased manufacturing costs. To overcome these limitations, research efforts have shifted towards alternative material systems, including 2D materials, III-V compound semiconductors, and carbon nanotubes (CNTs), which represent promising directions for TFET development. Among these, 2D-material-based TFETs have emerged as the most active research branch due to their atomically flat surfaces free of dangling bonds and excellent gate electrostatic control. The experimental results suggest that the use of heterojunction architectures can significantly reduce *SS* and enhance the on/off current ratio. Furthermore, integrating wide bandgap materials such as hexagonal boron nitride (hBN) with FNT properties has enabled the development of TFET-based memory devices, offering great potential for non-volatile applications. In particular, these memory-capable 2D TFETs are considered promising building blocks for future in-memory computing circuits based on non-von Neumann architectures. Materials such as monolayer MoS_2_, which is a direct-bandgap semiconductor, exhibit strong light absorption in the visible range, making them particularly suitable for optoelectronic and photodetection applications. On the fabrication side, significant progress has been made in the wafer-scale growth of single-crystal 2D semiconductors, laying a solid foundation for the future large-scale integration of 2D-TFETs [117]. III-V semiconductors, on the other hand, are compatible with current fabrication technologies and exhibit strong potential for TFET applications. However, further efforts are needed to address interface trap issues in order to reduce off-state leakage currents and improve the on/off current ratio. As for CNT-TFETs, advances in fabrication techniques and reliability design are essential to mitigate issues arising from impurities, oxidation, and associated degradation mechanisms [118].

Simulation-based studies primarily focus on optimizing TFET device architectures and selecting appropriate material systems. Techniques such as band engineering (QB-TFET) and alternative structural configurations like p-i-n-i TFETs can enhance tunneling rates. Dopingless (DL) or junctionless (JL) structures have also been proposed to eliminate the need for high doping concentrations, simplifying fabrication. With respect to the gate dielectric, the use of high-k dielectrics or negative capacitance effects can improve gate control and reduce SS. Nevertheless, it is critical to ensure the practical feasibility of proposed structures during simulation, as designs that significantly improve performance but are unrealizable in practice should be avoided.

In terms of practical applications, TFETs exhibit great promise across various domains. In the area of biosensing, most TFET-based applications remain at the simulation stage. When conducting such simulations, realistic considerations must be made—for instance, recessed structures should not be too narrow, as this may prevent biomolecules from entering the sensing region. Alternative device structures, such as ISFETs, may also be adopted to enhance practicality. At the circuit level, hybrid TFET-CMOS design has become one of the most actively explored directions. By leveraging the low subthreshold swing and low-power characteristics of TFETs while using MOSFETs to compensate for their shortcomings, hybrid designs—such as the 11T-SRAM architecture—have shown promising potential. Research by Kaifeng Wang et al. also provides valuable insights into process integration. However, compared to advanced FinFET/CMOS technologies, TFETs still face multiple challenges. For instance, circuit performance is highly sensitive to variations in TFET device parameters; process variability can significantly affect Ion, SS, delay, and power consumption, posing stringent requirements for circuit uniformity and yield control [119]. Additionally, in low-voltage design regimes, gate leakage through ultra-thin dielectrics and trap-assisted tunneling can lead to increased Ioff and degraded SS, thereby diminishing TFET effectiveness in ultra-low-power scenarios [120]. If these challenges can be adequately addressed, TFETs are poised for widespread adoption in the future.

In conclusion, the development of TFETs marks a transformative shift in addressing the physical limitations of traditional CMOS technology, particularly in achieving low power consumption, low operating voltage and better switching performance. Through extensive advancements in materials, including Si, Ge, III-V compounds and 2D materials, and structural innovations such as heterojunction and line tunneling, TFETs demonstrate the ability to surpass conventional limits of *SS* and power efficiency. The studies on novel TFET structures further exemplify the potential for performance enhancement of TFETs. Despite the challenges in uniform doping, interface stability, and process variability, ongoing research and simulation studies continue to optimize TFET performance and widen their application scope. TFETs hold substantial promise in areas such as digital circuits, sensors, and hybrid systems, paving the way for their role in next-generation electronic technologies. On the basis of the tremendous existing research results, coupled with continuous innovation in material science and fabrication processes, it is believed that, in the near future, TFETs will be essential for realizing their full potential in ultra-low-power, high-performance switching and sensing applications soon.
